# Pulmonary large cell carcinoma, highly positive for PD‐L1, shows marked response to pembrolizumab: A case report

**DOI:** 10.1111/1759-7714.13850

**Published:** 2021-02-19

**Authors:** Naoyuki Okabe, Hayato Mine, Hironori Takagi, Masayuki Watanabe, Satoshi Muto, Yuki Matsumura, Yutaka Shio, Hiroyuki Suzuki

**Affiliations:** ^1^ Department of Chest Surgery Fukushima Medical University School of Medicine Fukushima Japan

**Keywords:** immunotherapy, large cell carcinoma, lung, PD‐L1, pembrolizumab

## Abstract

Pulmonary large cell carcinoma (LCC) is classified as a poorly defined entity among non‐small cell lung cancers (NSCLCs). At present, there are no effective anticancer drugs, such as molecular targeted drugs, for LCC, and it has been reported that patient prognosis is poor. Recently, the development of immune checkpoint inhibitors (ICIs) has changed the therapeutic strategies for patients with NSCLC. Here, we present a case of LCC successfully treated with pembrolizumab. A 58‐year‐old man who was a former smoker was diagnosed with LCC. The postoperative stage was T3N2M0. During postoperative adjuvant chemotherapy, swelling of the supraclavicular lymph node was observed and the patient was diagnosed with recurrence. The patient was treated with two regimens of conventional cytotoxic chemotherapy; however, he experienced some hoarseness. Imaging confirmed swelling of the hilar and mediastinal lymph nodes and the patient was subsequently diagnosed with disease progression. Previous surgical specimens when immunostained showed that a high proportion of the tumor cells were positive for expression of programmed death‐ligand 1 (PD‐L1), and it was decided to commence treatment with pembrolizumab. This treatment resulted in rapid regression of the hilar and mediastinal lymph nodes, and a progression‐free period maintained for at least 24 treatment cycles. The patient's hoarseness improved, and the lymph nodes decreased in size. Immunotherapy targeting PD‐1/PD‐L1 may be an option for patients with PD‐L1 positive LCC. This case report suggests that treatment with ICIs may be important in the selection of treatment for not only LCC but also relatively rare NSCLC with high PD‐L1 expression.

## Graphical abstract

This report explains the therapeutic effect of immune checkpoint inhibitors (ICI) on large cell carcinoma (LCC). LCC is rare and there is no treatment target, so the prognosis is poor. As reported here, PD‐L1‐positive LCC showed a therapeutic effect following tratment with ICIs.

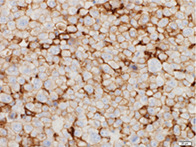

## INTRODUCTION

Pulmonary large cell carcinoma (LCC) is classified as a poorly defined entity among non‐small cell lung cancers (NSCLCs). At present, there are no effective anticancer drugs, such as molecular targeted drugs, for LCC and it has been reported that the prognosis is poor. Recently, the development of immune checkpoint inhibitors (ICIs) has changed the therapeutic strategies for NSCLCs. Here, we present a case of LCC successfully treated with pembrolizumab.

## CASE REPORT

**FIGURE 1:**
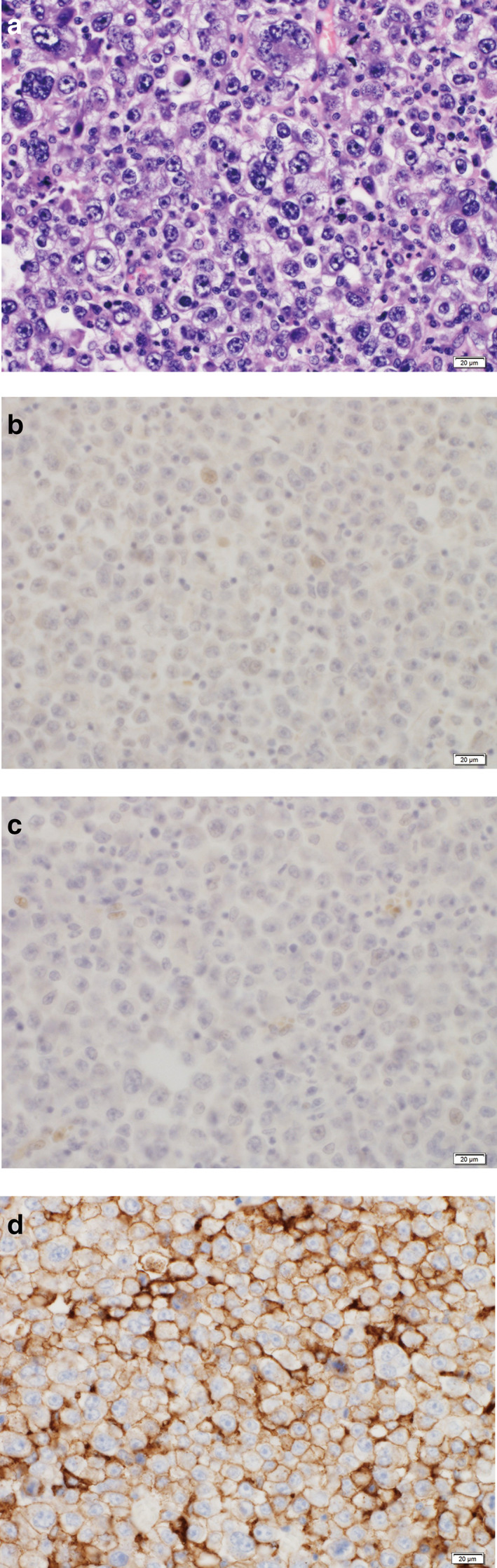
Lung surgical specimens. (a) Surgical specimens stained with hematoxylin and eosin (H&E). (b) Immunohistochemical staining for P40 was negative. (c) Immunohistochemical staining for thyroid transcription factor 1 (TTF 1) was negative. (d) A 22C3 IHC pharmDx assay detected PD‐L1 expression on about 100% of tumor cells

**FIGURE 2:**
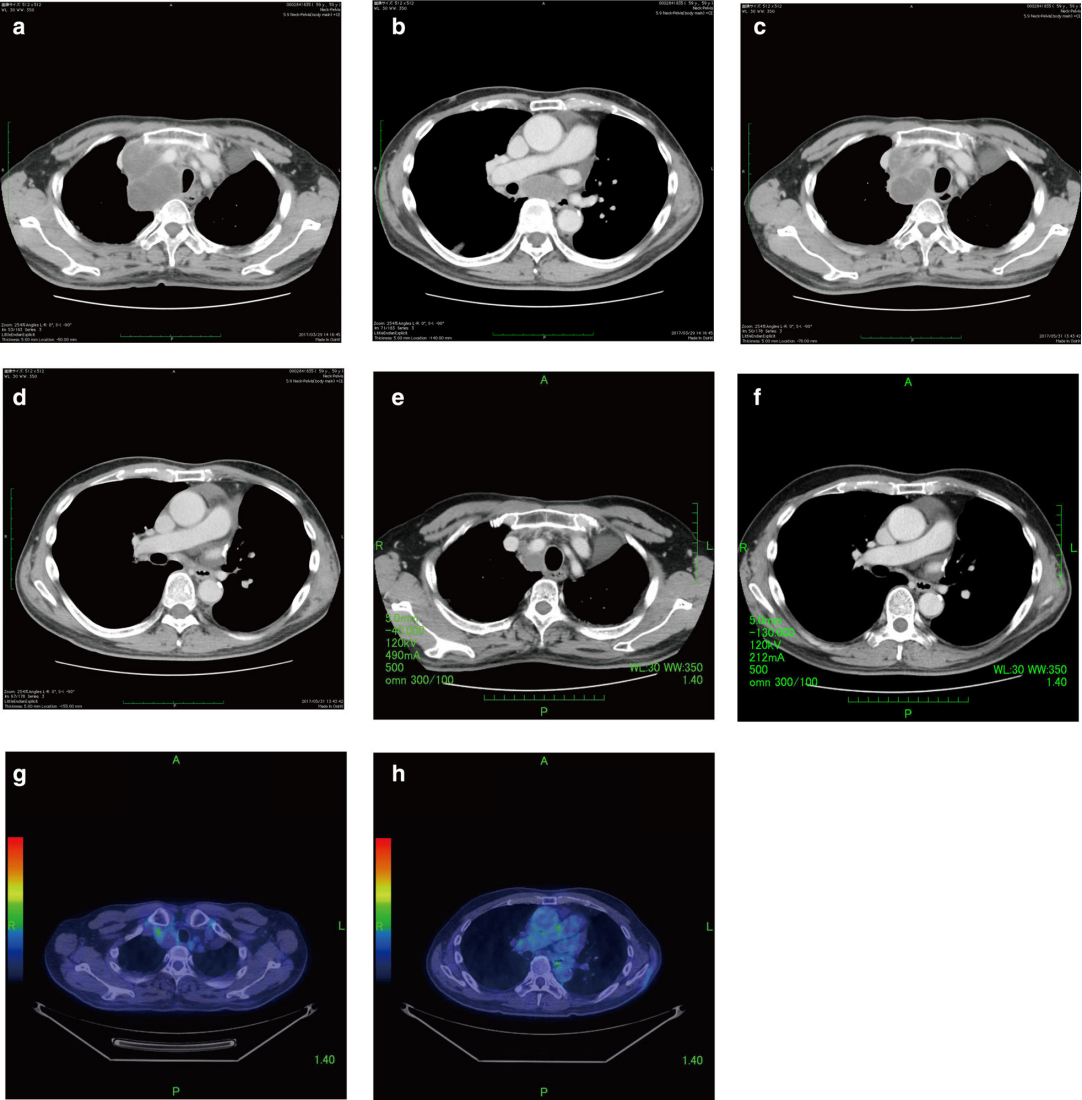
Clinical course: treatments, chest computed tomography (CT), and positron emission tomography (PET)‐CT. (a) Before pembrolizumab administration, CT shows superior mediastinal lymph node swelling. (b) Before pembrolizumab administration, CT shows lower mediastinal lymph node swelling. (c) After three cycles of pembrolizumab, CT shows reduction of the superior mediastinal lymph node. (d) After three cycles of pembrolizumab, CT shows reduction of the lower mediastinal lymph node. (e) After 24 cycles of pembrolizumab, CT shows reduction of the superior mediastinal lymph node. (f) After three cycles of pembrolizumab, CT shows reduction of the lower mediastinal lymph node. (g) PET‐CT 24 cycles after pembrolizumab shows a pronounced decrease in tracer uptake in the superior mediastinal lymph node. (h) PET‐CT 24 cycles after pembrolizumab shows a pronounced decrease in tracer uptake in the lower mediastinal lymph node

A 58‐year‐old man who was a former smoker was referred to our hospital with a history of a prolonged cough and bloody sputum. After careful examination, he was diagnosed with non‐small cell lung cancer (NSCLC) and subsequently underwent radical surgical resection. The tumor was confirmed as p‐T3N2M0 pulmonary large cell carcinoma (P40‐negative and thyroid transcription factor 1‐negative) (Figure 1a–c). There were no genetic abnormalities that could be targeted for therapy in this patient. During postoperative adjuvant chemotherapy (CDDP + VNR), swelling of the supraclavicular lymph node was observed, and the patient was diagnosed with a recurrence. He was treated with two regimens of conventional chemotherapy (CDDP + PEM + BEV, CDDP + ETP). However, he developed hoarseness, and imaging showed swollen mediastinal lymph nodes (Figure 2a,b). Using previous surgical specimens, a 22C3 immunohistochemical (IHC) pharmDx assay detected programmed death‐ligand 1 (PD‐L1) expression in about 100% of tumor cells (Figure 1d). We diagnosed postoperative recurrence and decided to administer pembrolizumab because of the involvement of multiple lymph nodes and the difficulty in controlling the disease with radiation. After starting treatment with pembrolizumab, his hoarseness immediately improved. Treatment with pembrolizumab resulted in rapid regression of the mediastinal and supraclavicular lymph nodes. Computed tomography (CT) and positron emission tomography (PET‐CT) scan showed shrunken lymph nodes, and a partial response was noted (Figures 2c–f). The patient remained progression‐free for 24 cycles of treatment. Pembrolizumab was continued for 24 cycles without serious adverse events or tumor progression. Immunotherapy targeting PD‐1/PD‐L1 may be an option for patients with PD‐L1‐positive LCC.

## DISCUSSION

Here, we report a rare case of LCC that was successfully treated with pembrolizumab. LCC has been reported to have a higher rate of PD‐L1 expression compared to other NSCLCs. Therefore, ICIs may be effective against such PD‐L1‐positive LCCs.^1^

Historically, LCC has accounted for approximately 10% of NSCLCs, and LCC is the third most common subtype of NSCLC after adenocarcinoma and squamous cell carcinoma. The 2015 WHO Classification of Lung Tumors made major changes to the classification of LCC, emphasizing the significance of immunophenotyping in tumor classification. The new WHO classification restricts the diagnosis of LCC to “resected tumors that lack any clear morphological or immunohistochemical differentiation towards small cell carcinoma, adenocarcinoma or squamous cell carcinoma.”^2^ Only those with null or unclear immunophenotypes are currently classified as LCC.^1^ Therefore, LCC now accounts for approximately 7.5% of all lung cancers which makes them one of the rarest subtypes of NSCLC. In addition, there is no specific molecular targeting drug for large cell lung cancer, and LCC is reported to have a poor prognosis.^2^, ^3^

The results of the KEYNOTE‐024 trial and KEYNOTE‐042 trials showed that pembrolizumab, a monoclonal antibody against PD‐1, was more effective than conventional platinum‐based combination chemotherapy as a first‐line treatment for advanced NSCLC expressing PD‐L1 on the tumor cells, which are negative for epidermal growth factor receptor gene (EGFR) mutation and anaplastic lymphoma kinase gene (*ALK*) rearrangement.^4^, ^5^ Furthermore, PD‐L1 inhibitors have been reported to be effective in rare classifications of NSCLC, such as LCC and pleomorphic carcinoma.^6^, ^7^, ^8^

It has been suggested that LCC tends to have a higher rate of PD‐L1 positivity compared to other NSCLCs. The reported PD‐L1–positivity in LCC was 81% at TPS 1% or greater cutoff and 47% at 50% or greater cutoff.^1^ As reported by other studies, PD‐L1 expression was found to be associated with tumor grade.^9^, ^10^ Chan et al. reported that LCCs did not express known target molecules for adenocarcinoma or squamous cell carcinoma but PD‐L1 and cell cycle regulatory genes were frequently expressed.^1^ In the current study, the patient was diagnosed with LCC using IHC. Conventional chemotherapy was found to be ineffective, but PD‐L1 was highly expressed. We chose to treat the patient with an ICI and found a remarkable therapeutic effect. Given that LCC is relatively rare among NSCLCs, with no targetable genetic abnormality or effective chemotherapy, and a higher expression of PD‐L1 compared to other NSCLCs, we believe that ICIs may be an important treatment option for LCC.

ICIs targeting PD‐L1 have been shown to be effective against multiple cancer types, including NSCLC.^4^ Recently, there have been reports that ICI treatment is effective against rare histological types of NSCLC where PD‐L1 is highly expressed.^6^, ^7^, ^8^ This case report suggests the possibility of a new treatment strategy for LCC, which is generally thought to have a poor prognosis.

## CONFLICT OF INTEREST

The authors declare no conflicts of interest associated with this manuscript.
